# The antioxidant effects of the flavonoids rutin and quercetin inhibit oxaliplatin-induced chronic painful peripheral neuropathy

**DOI:** 10.1186/1744-8069-9-53

**Published:** 2013-10-23

**Authors:** Maria Isabel Azevedo, Anamaria Falcão Pereira, Ricardo Braz Nogueira, Flávio Esmeraldo Rolim, Gerly AC Brito, Deysi Viviana T Wong, Roberto CP Lima-Júnior, Ronaldo de Albuquerque Ribeiro, Mariana Lima Vale

**Affiliations:** 1 Medical Sciences Post-Graduation, Department of Clinical Medicine, Faculty of Medicine, Federal University of Ceará, Fortaleza, Brazil; 2 Department of Physiology and Pharmacology, Federal University of Ceará, Fortaleza, CE, Brazil; 3 Department of Morphology, Federal University of Ceará, Fortaleza, Brazil

**Keywords:** Oxaliplatin, Oxidative stress, Pain, Flavonoids, Neuropathy

## Abstract

**Background:**

Oxaliplatin, the third-generation platinum compound, has evolved as one of the most important therapeutic agents in colorectal cancer chemotherapy. The main limiting factor in oxaliplatin treatment is painful neuropathy that is difficult to treat. This side effect has been studied for several years, but its full mechanism is still inconclusive, and effective treatment does not exist. Data suggest that oxaliplatin’s initial neurotoxic effect is peripheral and oxidative stress-dependent. A spinal target is also suggested in its mechanism of action. The flavonoids rutin and quercetin have been described as cell-protecting agents because of their antioxidant, antinociceptive, and anti-inflammatory actions. We proposed a preventive effect of these agents on oxaliplatin-induced painful peripheral neuropathy based on their antioxidant properties.

**Methods:**

Oxaliplatin (1 mg/kg, i.v.) was injected in male Swiss mice, twice a week (total of nine injections). The development of sensory alterations, such as thermal and mechanical allodynia, was evaluated using the tail immersion test in cold water (10°C) and the von Frey test. Rutin and quercetin (25-100 mg/kg, i.p.) were injected 30 min before each oxaliplatin injection. The animals’ spinal cords were removed for histopathological and immunohistochemical evaluation and malondialdehyde assay.

**Results:**

Oxaliplatin significantly increased thermal and mechanical nociceptive response, effects prevented by quercetin and rutin at all doses. Fos immunostaining in the dorsal horn of the spinal cord confirmed these results. The oxidative stress assays mainly showed that oxaliplatin induced peroxidation in the spinal cord and that rutin and quercetin decreased this effect. The flavonoids also decreased inducible nitric oxide synthase and nitrotyrosine immunostaining in the dorsal horn of the spinal cord. These results suggest that nitric oxide and peroxynitrite are also involved in the neurotoxic effect of oxaliplatin and that rutin and quercetin can inhibit their effect in the spinal cord. We also observed the preservation of dorsal horn structure using histopathological analyses.

**Conclusions:**

Oxaliplatin induced painful peripheral neuropathy in mice, an effect that was prevented by rutin and quercetin. The mechanism of action of oxaliplatin appears to be, at least, partially oxidative stress-induced damage in dorsal horn neurons, with the involvement of lipid peroxidation and protein nitrosylation.

## Background

Platinum compounds represent an important antitumor drug class that is widely used in the treatment of several solid human tumors. The first synthesized molecule was cisplatin (1854) and afterward carboplatin. Oxaliplatin is a third-generation platinum agent with significant cytotoxic activity that is different from other platinum agents, with diminished antitumoral resistance [1,2]. Today it is used in combination with 5-fluorouracil (5-FU) and leucovorin (LV) in the FOLFOX protocol, one of the first-line treatment regimen against metastatic colorectal cancer [3]. The inclusion of oxaliplatin in the FOLFOX protocol has improved response rate, time to disease progression, and overall survival for patients with this type of cancer [4-6].

Notwithstanding this progress in antitumor therapy, side-effects have increased [7]. The toxicity profile, including neutropenia, nephrotoxicity, and ototoxicity are observed but in a significantly lower rate when compared with the peripheral sensory neuropathy, which is the main limiting adverse event in oxaliplatin treatment. This neurotoxic effect appears as transient, acute, sensitive neuropathy, the symptoms of which include perioral and distal paresthesias, cumulative dose-related sensitive neuropathy [2], distal limbs paresthesias, and dysesthesias that affect daily activities and impair the patient’s quality of life.

Joseph et al. [8] showed that oxaliplatin induced acute neuropathy beginning with peripheral nociceptive fiber injury caused by oxidative stress, and these effects are prevented with antioxidant agents. Adequate treatment is usually difficult to achieve and mostly disappointing because no fully effective drug yet exists. Preventive therapies include ionic calcium and magnesium infusions, vitamin E, glutathione, glutamine, and *N*3acetylcysteine, with the aim of reducing neuropathic symptoms in patients who undergo antitumor therapy [9,10]. Symptomatic treatment is based on neuromodulating agents. Once neuropathic pain develops, common analgesics are ineffective. The therapy tends to use adjuvant drugs, such as anticonvulsants (e.g., gabapentin, carbamazepine), local anesthetics (e.g., lidocaine), opioids (e.g., tramadol, morphine), tricyclic antidepressants (e.g., amitryptyline), and selective serotonin reuptake inhibitors [11].

Recently, new hypotheses have been proposed to explain the oxaliplatin-related neuronal injury. A neuronal subpopulation of spinal cord dorsal ganglia appears to be directly injured by oxaliplatin, causing neural atrophy without any significant loss of cells [12]. In a recent study, Joseph and co-workers [8] showed that oxaliplatin causes pain by an action on a subset of nociceptors, the IB4-positive DRG neurons. Oxidative stress has also been suggested to be the direct cause of neuronal injury. Acute thermal and mechanical hyperalgesia caused by a single intravenous dose of oxaliplatin was inhibited by antioxidant agents, such as vitamin C and L-carnitine [8], but this previous study was performed with only a single oxaliplatin dose. Chronic cumulative oxaliplatin damage has not been proven to follow the same pattern, but this may be a key focus of future research.

The flavonoids rutin and quercetin are polyphenolics compounds found in vegetables, fruits, herbs, leaves, seeds [13,14], red wine, tea, coffee, beer, and several medicinal plants. Rutin is a bioflavonoid and antioxidant. It is water-soluble and converted to quercetin once it enters the blood stream [15]. Several studies have found that these flavonoids have antiinflammatory [16-18], analgesic [19], and antioxidant [20] effects.

Based on this background evidence on the biological effects of flavonoid compounds and the possible oxidative-related mechanism of neuronal injury due to oxaliplatin injection, the present study investigated the neuroprotective effects of rutin and quercetin on oxaliplatin-induced neurotoxicity.

## Results

### Decrease in mechanical and cold nociceptive threshold induced by oxaliplatin

**Figure 1:**
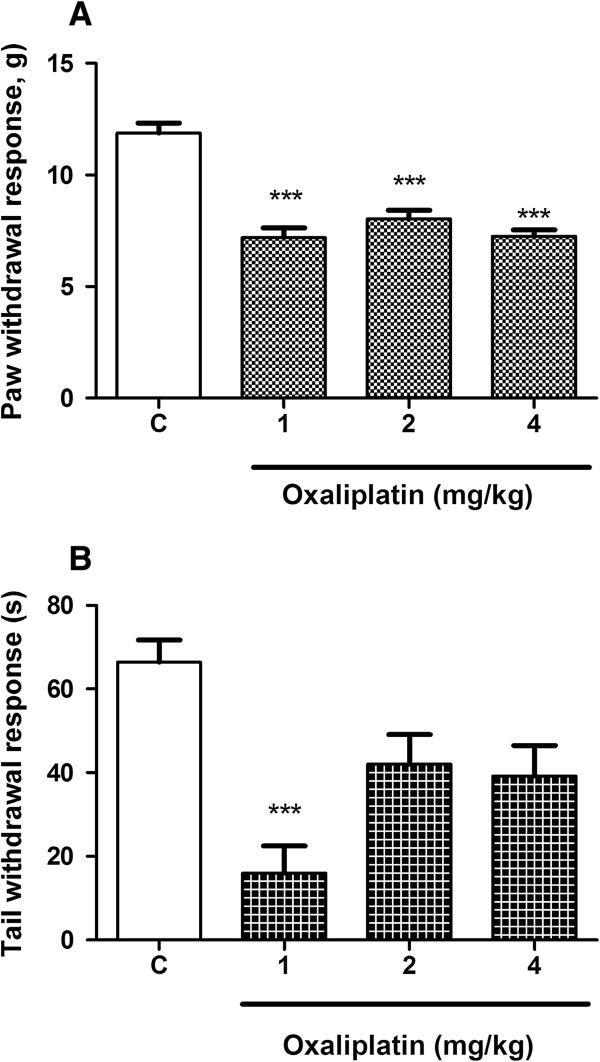
**Decrease of mechanical and cold nociceptive threshold induced by oxaliplatin.** Each mouse received two intravenous injections per week for 4,5 weeks, totalizing nine injections. Withdrawal responses were determined once a week until the 49th day. Panel **A**: paw withdrawal threshold of control (C, vehicle, n = 12) and treated (oxaliplatin 1 mg/kg, 2 mg/kg and 4 mg/kg, n = 12) mice to the electronic pressure meter (electronic von Frey) applied to the plantar surface of the hind paw. Panel **B**: Tail withdrawal threshold of Control (C, vehicle, n = 12) and treated (oxaliplatin 1 mg/kg, 2 mg/kg and 4 mg/kg, n = 12) mice to tail immersion in cold non-noxious (10°C) water. The results are reported as the means ± SEM paw withdrawal threshold (g) for mechanical threshold (panel **A**) and tail withdrawal response (s) for the cold threshold (panel **B**). A significant reduction in mechanical and thermal threshold (***p < 0.001, ANOVA followed by Student Newman-Keuls *post hoc* test), was observed when compared with the vehicle group.

Mechanical nociceptive threshold was evaluated in the hind paws of mice that received intravenous injections of oxaliplatin (1, 2 and 4 mg/kg) and the cold non-noxious threshold (10°C) was evaluated using the tail immersion test in mice that received oxaliplatin at the same doses. The nociceptive threshold of mice was assessed, before and after oxaliplatin injections, every week and it was observed a decrease of nociceptive threshold for mechanical and thermal stimuli. The decrease in mechanical and cold nociceptive threshold developed by day 28 and was evaluated until 56th day. Figure 1A shows the decrease of mechanical nociceptive threshold of mice at the 49th day after oxaliplatin first injection, which seems to be the period in which oxaliplatin reaches the maximum of effect. We observed a significant (p < 0.001) decrease in the nociceptive threshold (7.19 ± 0.44, 1 mg/kg) in all the doses tested (without no differences between them) when compared to the control group (11.88 ± 0.44). The Figure 1B shows the decrease of cold threshold of mice at the 49th day after oxaliplatin first injection. A decrease in the withdrawal threshold was observed with all the doses, but only the dose of 1 mg/kg reached a significant difference (16.0 ± 6.5, p < 0.001), when compared to control group (66.43 ± 5.2). For this reason we choose this dose to perform the following experiments.

### Effect of rutin and quercetin upon the mechanical nociceptive threshold in mice subjected to oxaliplatin-induced painful peripheral neuropathy

**Figure 2:**
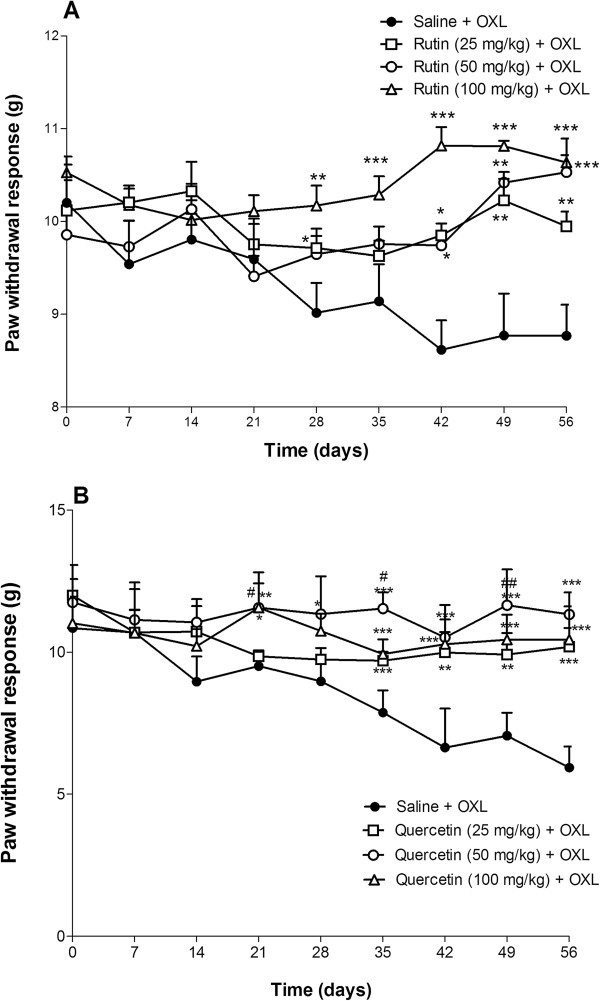
**Antinociceptive effects of rutin and quercetin on oxaliplatin-induced mechanical nociceptive threshold decrease (von Frey).** The mice received two intravenous injections of oxaliplatin (OXL; 1 mg/kg) per week, for 4.5 weeks for a total of nine injections. Rutin **(A)** and quercetin **(B)** were injected intraperitoneally 30 min before every OXL administration. The control group received saline instead of rutin or quercetin. Mechanical threshold was assessed before and every 7 days after each treatment. The data points represent the mean ± SEM the paw withdrawal response in grams (g) in six animals.*p < 0.05, **p < 0.01, ***p < 0.001 (ANOVA followed by Student Newman-Keuls *post hoc* test).

Mechanical nociceptive threshold was evaluated in the hind paws of mice that received an intravenous injection of oxaliplatin (1 mg/kg). Both rutin and quercetin inhibited the oxaliplatin-induced decrease in mechanical nociceptive threshold. Rutin, at all doses tested, prevented the nociceptive response beginning on day 28, exerting a more pronounced effect (*p* < 0.05) at a dose of 100 mg/kg on day 42 of treatment (10.82 ± 0.20, Figure 2A) versus oxaliplatin control group (8.61 ± 0.31). Quercetin also inhibited at all doses tested the effect of oxaliplatin beginning on day 21 and exerting a more pronounced effect, at a dose of 50 mg/kg, on day 56 of treatment (10.53 ± 0.18, Figure 2B) compared to the oxaliplatin control group (8.77 ± 0.33).

### Effect of rutin and quercetin upon cold nociceptive threshold in mice subjected to oxaliplatin-induced painful peripheral neuropathy

**Figure 3:**
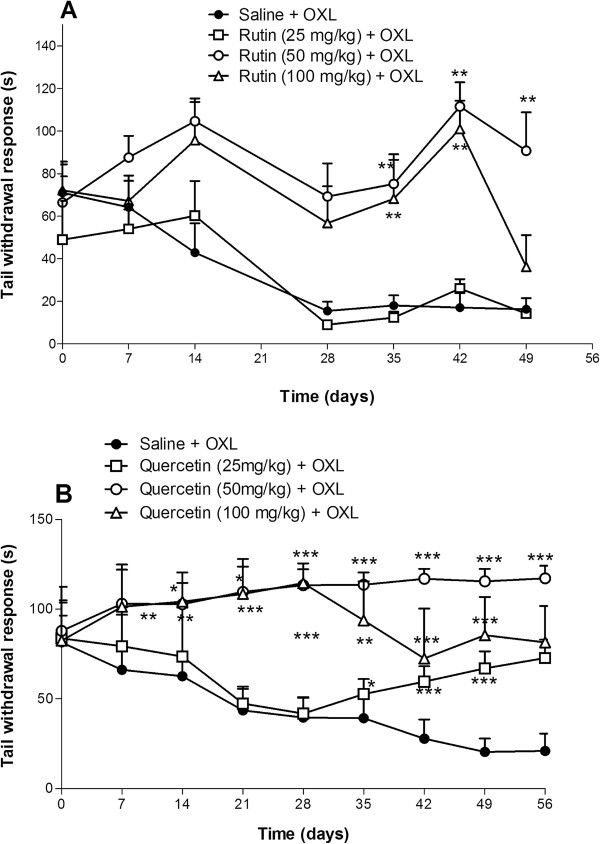
**Antinociceptive effects of rutin and quercetin on cold nociceptive threshold in oxaliplatin (OXL)-treated mice (tail immersion).** The mice received two intravenous injections of OXL (1 mg/kg) per week for 4.5 weeks for a total of nine OXL injections. Rutin **(A)** and quercetin **(B)** were injected intraperitoneally 30 min before every OXL administration. The control group received saline instead of rutin or quercetin. The thermal test (tail immersion test in cold water, 10°C) was conducted before and every 7 days after each treatment. The data points represent the mean ± SEM of the reaction time in seconds after tail immersion in cold water in six animals.*p < 0.05, **p < 0.01, ***p < 0.001 (ANOVA followed by Student Newman-Keuls*post hoc* test).

The threshold for cold non-noxious (10°C) was evaluated using the tail immersion test in mice that received an intravenous injection of oxaliplatin (1 mg/kg). Oxaliplatin increased the tail withdrawal response, and both rutin and quercetin prevented this effect. Rutin, only at doses of 50 and 100 mg/kg, had a significant inhibitory effect, reaching a maximum effect (111.50 ± 11.46, *p* < 0.01) at a dose of 50 mg/kg on day 42, compared with the control group (17.00 ± 7.27, Figure 3A). Quercetin, at all doses, prevented the decrease in cold threshold (*p* < 0.001) beginning on day 7, with a maximal response on day 49 at the dose of 50 mg/kg (117.16 ± 2.83) versus control group (21.00 ± 4.27, Figure 3B).

### Histopathological analysis of the dorsal horn of the spinal cord in mice subjected to oxaliplatin-induced painful peripheral neuropathy and treated with rutin and quercetin

**Figure 4:**
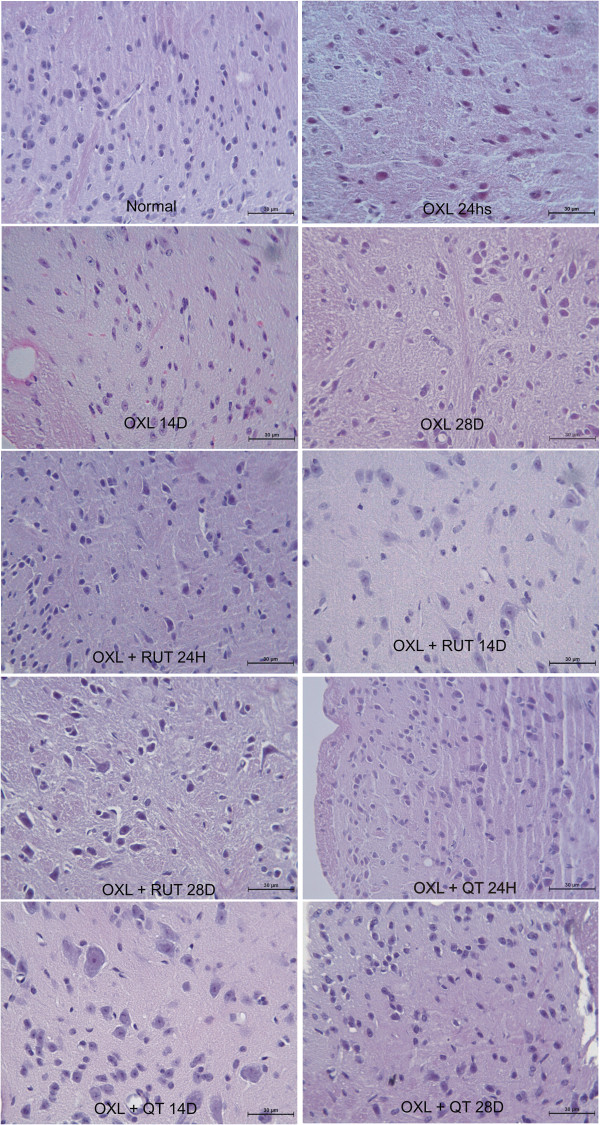
**Photomicrographs of the dorsal horn of the spinal cord of mice subjected to oxaliplatin (OXL)-induced neurotoxicity and treated with rutin (RUT) or quercetin (QT).** The mice received two intravenous injections of OXL (1 mg/kg) per week for 4.5 weeks for a total of nine OXL injections. Rutin and quercetin (50 mg/kg) were injected intraperitoneally 30 min before every OXL administration. The control group received saline instead of rutin and quercetin. The figure shows hematoxylin-eosin staining (400× magnification).

The histopathological analysis revealed differences between naive and oxaliplatin-treated mice. Twenty-four hours after oxaliplatin injection, the presence of discrete lacunar spaces was observed between neuronal cells, suggesting nervous tissue edema. We also observed shrinkage of the size of dorsal horn neurons, mainly from the 14th experimental day, suggesting the atrophy of neurons and increase in glial cell number. Both rutin and quercetin prevented these changes, maintaining the tissue with aspects similar to the naive group (Figure 4).

### Histopathological analysis of skin harvested from the paws of mice subjected to oxaliplatin-induced painful peripheral neuropathy and treated with rutin and quercetin

**Figure 5:**
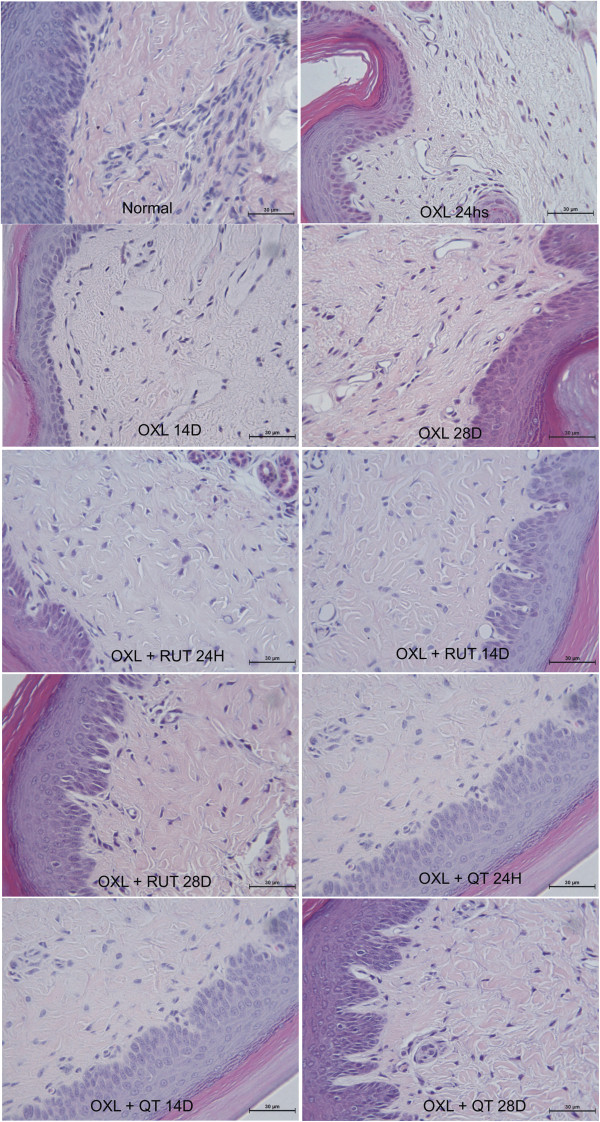
**Photomicrographs of the skin harvested from the paws of mice subjected to oxaliplatin (OXL)-induced neurotoxicity and treated with rutin (RUT) or quercetin (QT).** The mice received two intravenous injections of OXL (1 mg/kg) per week for 4.5 weeks for a total of nine OXL injections. Rutin and quercetin (50 mg/kg) were injected intraperitoneally 30 min before every OXL administration. The control group received saline instead of rutin and quercetin. The figure shows hematoxylin-eosin staining (400× magnification).

The histopathological analysis of the paw skin of mice injected with oxaliplatin showed the presence of little lacunar spaces between collagen fibers, suggesting discrete edema of conjunctive tissue, without any other sign of inflammation. This edema appeared to be most evident at the first week of oxaliplatin treatment. Both rutin and quercetin prevented the development of this event (Figure 5).

### Effects of rutin and quercetin on oxaliplatin-induced lipid peroxidation, reflected by malondialdehyde (MDA) levels

**Figure 6:**
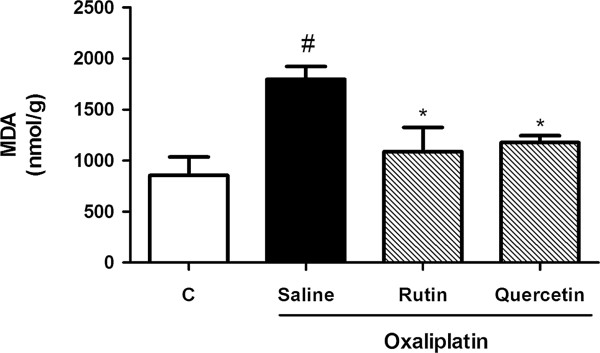
**Effect of rutin and quercetin on malondialdehyde (MDA) levels in the spinal cord.** The mice received two intravenous injections of OXL (1 mg/kg) per week for 4.5 weeks for a total of nine OXL injections. Rutin and quercetin (50 mg/kg) were injected intraperitoneally 30 min before every OXL administration. The control group received saline instead of rutin and quercetin. Fourteen days after the first dose, a portion of the spinal cord was collected and processed to measure MDA levels. The results are expressed as mean ± SEM. *p < 0.05, compared with saline plus oxaliplatin-treated group; ^#^*p* < 0.05, compared with naïve (C) group (ANOVA followed by Student Newman-Keuls *post hoc* test).

Figure 6 shows that oxaliplatin significantly increased (by 110.1%, *p* < 0.05) MDA levels in spinal cord samples compared with control mice. This effect was prevented (*p* < 0.05) by both rutin (65.1%) and quercetin (52.7%).

### Immunohistochemical analysis of the dorsal horn of the spinal cord in mice subjected to oxaliplatin-induced painful peripheral neuropathy and treated with rutin and quercetin

**Figure 7:**
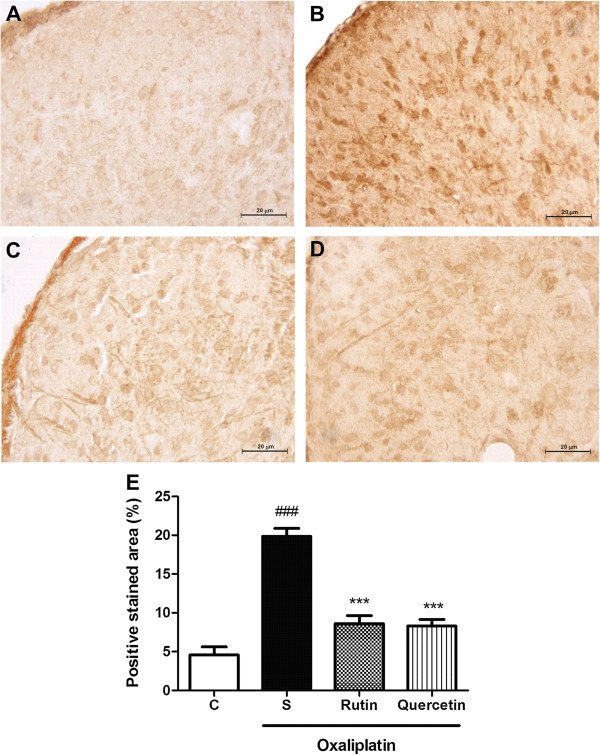
**Fos immunostaining in the dorsal horn of the spinal cord in mice subjected to oxaliplatin (OXL)-induced neurotoxicity and treated with rutin or quercetin.** The mice received two intravenous injections of OXL (1 mg/kg) per week for 4.5 weeks for a total of nine OXL injections. Rutin or quercetin (50 mg/kg) were injected intraperitoneally 30 min before every OXL administration. The control group received saline instead of rutin and quercetin. **(A)** Naive animals; **(B)** Oxaliplatin plus saline; **(C)** Oxaliplatin plus rutin (50 mg/kg); **(D)** Oxaliplatin plus quercetin (50 mg/kg). 400× magnification. **(E)** Bars show the percentage of positive Fos staining area, mean ± SEM (n = 4). ***p < 0.001, compared with saline plus oxaliplatin-treated group (S); ^###^*p* < 0.001, compared with naive (C) group (ANOVA followed by Student Newman-Keuls *post hoc* test).

**Figure 8:**
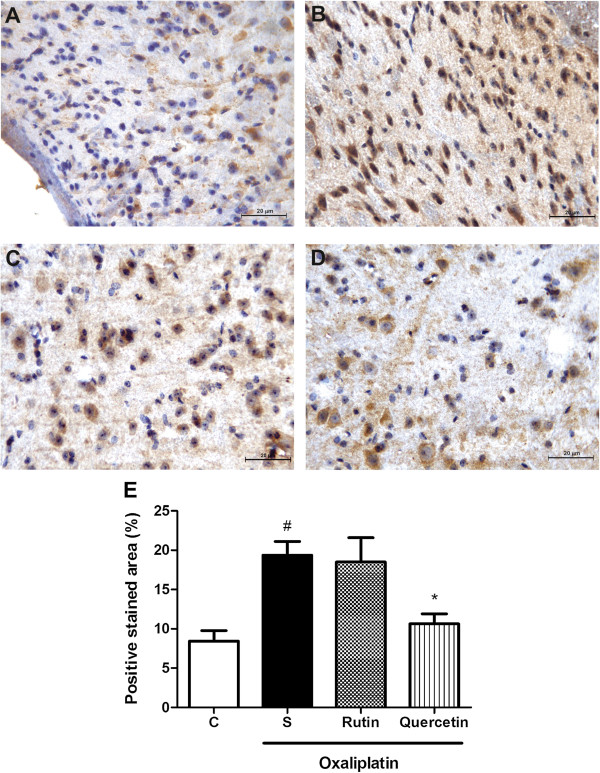
**Nitrotyrosine immunostaining in the dorsal horn of the spinal cord of mice subjected to oxaliplatin (OXL)-induced neurotoxicity and treated with rutin or quercetin.** The mice received two intravenous injections of OXL (1 mg/kg) per week for 4.5 weeks for a total of nine OXL injections. Rutin or quercetin (50 mg/kg) were injected intraperitoneally 30 min before every OXL administration. The control group received saline instead of rutin and quercetin. **(A)** Naive animals; **(B)** Oxaliplatin plus saline; **(C)** Oxaliplatin plus rutin (50 mg/kg); **(D)** Oxaliplatin plus quercetin (50 mg/kg). 400× magnification. **(E)** Bars show the percentage of positive nitrotyrosine staining area, mean ± SEM (n = 4). *p < 0.05, compared with saline plus oxaliplatin-treated group (S); ^#^*p* < 0.05, compared with naive (C) group (ANOVA followed by Student Newman-Keuls *post hoc* test).

**Figure 9:**
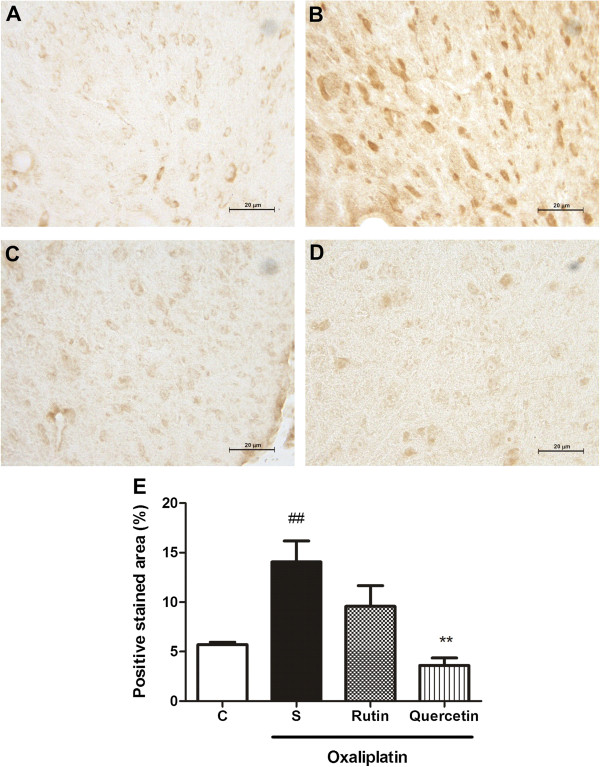
**iNOS immunostaining in the dorsal horn of the spinal cord of mice subjected to oxaliplatin (OXL)-induced neurotoxicity and treated with rutin or quercetin.** The mice received two intravenous injections of OXL (1 mg/kg) per week for 4.5 weeks for a total of nine OXL injections. Rutin or quercetin (50 mg/kg) were injected intraperitoneally 30 min before OXL administration. The control group received saline instead of rutin and quercetin. **(A)** Naive animals; **(B)** Oxaliplatin plus saline; **(C)** Oxaliplatin plus rutin (50 mg/kg); **(D)** Oxaliplatin plus quercetin (50 mg/kg). 400× magnification. **(E)** Bars show the percentage of positive iNOS staining area, mean ± SEM (n = 4). **p < 0.01, compared with saline plus oxaliplatin-treated group (S); ^##^*p* < 0.01, compared with naive (C) group (ANOVA followed by Student Newman-Keuls *post hoc* test).

The immunohistochemical analysis revealed an increase in Fos, nitrotyrosine, and inducible nitric oxide synthase(iNOS) immunoexpression, characterized by brown-colored cells in the dorsal horn of the spinal cord in mice subjected to oxaliplatin-induced painful neuropathy (Figures 7,8,9). The Fos immunohistochemical analysis showed intense staining (*p* < 0.001) of neuronal cells, especially in the nucleus region compared with naive animals (Figure 7B and E). Both rutin (Figure 7C) and quercetin (Figure 7D) significantly inhibited this effect (*p* < 0.001; Figure 7E). The nitrotyrosine immunohistochemical analysis showed intense staining (*p* < 0.05; Figure 8E) of neuronal cells, especially at the cytoplasmic level (Figure 8B) compared with the naive group (Figure 8A). Quercetin (Figure 8D) but not rutin (Figure 8C) significantly inhibited this effect (*p* < 0.05; Figure 8E). The iNOS immunohistochemical analysis showed intense staining (*p* < 0.01; Figure 9E) of neuronal cells, especially at the cytoplasmic level (Figure 9B) compared with the naive group (Figure 9A). Quercetin (Figure 9D) but not rutin (Figure 9C) significantly inhibited this effect (*p* < 0.01; Figure 9E).

## Discussion

Painful peripheral neuropathy appears to be caused by all platinum compounds used in cancer chemotherapy. Among these compounds, the third-generation drug oxaliplatin or the oxaliplatin-based regiments are one of the first choices in the treatment of colorectal cancer, but it is associated with the development of acute and chronic painful neuropathy that is difficult to treat. The acute phase affects almost every patient who receives this drug, and the chronic effects of oxaliplatin are cumulative and limit treatment. This side effect of oxaliplatin has been studied for several years, but its full mechanism of action is still inconclusive, and effective treatment does not exist. The present study proposed preventive and therapeutic effects of the flavonoids rutin and quercetin on painful peripheral neuropathy induced by oxaliplatin.

Previous work by Ling et al. [21] showed that rats treated with oxaliplatin developed painful and long-lasting hyperalgesia in mechanical and thermal tests. They used doses of 1, 2, and 4 mg/kg oxaliplatin with repeated intravenous administration. Based on that study, we tested the same doses in mice, also using a mechanical test and non-noxious cold thermal test (i.e., tail immersion test), and we found that oxaliplatin at 1 mg/kg was the lowest effective dose which was consistently capable of producing nociception in all the tests employed in this study. In a review published by Le Bars and colleagues [22] the influence of species and animal genetic line on nociception is discussed. They point out the clear existence of intraspecific and interspecific variability concerning pharmacokinetics and pharmacodynamics of substances in nociceptive tests. It could in part explain the differences found between our results, in mice, and that from Ling et al. [21], whose experiments were performed in rats, in regard to the dose of oxaliplatin used to induce peripheral neurophathy. Therefore, we chose the 1 mg/kg dose of oxaliplatin to investigate the effects of flavonoids on the oxaliplatin-related nociception.

In the behavioral experiments, we showed that both rutin and quercetin inhibited the decrease in mechanical nociceptive threshold in the hind paws of mice subjected to this neuropathy. This inhibitory effect was also observed in cold threshold in the tail immersion test. We found that quercetin exerted a better effect than rutin.

Fos is the protein product of the immediate-early gene c-Fos which is found in neuronal nuclei. In most neurons, Fos levels are low under basal conditions, but only changes in afferent inputs and / or changes in external stimuli, which induce c-fos expression, result in phenotypic reprogramming of the cell (Reviewed by [23]). Therefore, Fos is a useful marker to trace the effects of pharmacological, electrical, and physiological stimuli in the nervous system, and it has been used to indicate pain and neuroplasticity [24-26]. Understanding this, we performed Fos immunostaining in dorsal horn neurons. The results supported the hypothesis that oxaliplatin activates nociceptive pathways because dorsal horn neurons exhibited greater Fos expression in the oxaliplatin-treated group. In addition, rutin and quercetin inhibited Fos expression, suggesting an antinociceptive effect of these flavonoids that corroborates the behavioral results.

The histopathological changes in the dorsal horn area were also prevented by rutin and quercetin, with a better effect of quercetin. The flavonoids prevented the shrinkage of neurons after 14 days and light edema formation that occurred beginning on day 1. Edema occurred in hind paw conjunctive tissue and was prevented by flavonoid treatment. Other authors showed that another platinum compound, cisplatin, causes neuronal nuclear DNA damage and mitochondrial vacuolization [27-29]. Another study found morphological changes at 144 h of cisplatin treatment, including a decrease in the size of neuronal cells and extensive mitochondrial vacuolization throughout the cell body, but the nuclear membrane remained intact [29]. It is known that neurotoxicity leads to an excessive neuronal activity leading to an increased glutamate release. Repetitive nociceptive stimulation and pathologic states resulting in glutamate release, therefore, may lead to changes in extracellular space volume and architecture, affecting volume transmission, enhancing glutamate neurotoxicity and neuronal damage [30]. In fact, we have highlighted the increased neuronal activity in the dorsal horn of oxaliplatin injected animals through c-Fos expression, which was prevented by rutin and quercetin treatment.

Recent work has discussed the differences between oxaliplatin- and paclitaxel-induced peripheral painful neuropathy. Oxaliplatin promoted mitochondrial changes, and chemotherapy-induced mitochondrial injury may be the event that leads to mechanical and thermal nociceptive alteration. Additionally, mitochondrial dysfunction led to an increase in the release of free electrons from the electron transport system and subsequent oxidative and nitrative stress in peripheral neuronal cells [31].

Oxidative stress was also suggested by Joseph et al. as responsible for the peripheral neuronal injury caused by oxaliplatin. They showed that acute painful peripheral neuropathy induced by oxaliplatin appears to be mediated by an action on the IB4-positive nociceptors [8]. These authors also suggested that the nontoxic intervention with antioxidants could be useful to treat the oxaliplatin-related toxicity. The peripheral edema of the hind paws observed in the present study may be partially explained by this previous work. We investigated the effects of compounds with known antioxidant properties on this effect of oxaliplatin. In our study, a remarkable protective effect was observed. Our findings reinforce the hypothesis that the oxidative stress event leads to chronic neuropathy due to the oxaliplatin injection.

Recently a cellular model of oxaliplatin neurotoxicity based on redox unbalance as mechanism of damage has been proposed [32] and the antioxidant effect silibilin and alpha-tocopherol [33].

The flavonoids are compounds with antioxidant properties, in addition to their antiinflammatory [16-18] and antinociceptive [34,35] effects. The flavonoids cyanidin and quercetin have antioxidant potential that is four-fold greater that of the vitamin E analog Trolox. Quercetin, through the blockade of reactive oxygen species (ROS) production, protects mouse hippocampal cells from oxidative toxicity and lipid peroxidation induced by glutamate. It also has an inhibitory effect on oxidative stress-induced neurotoxicity that is exerted through three distinct mechanisms: a direct effect on GSH metabolism, an antioxidant effect, and the maintenance of low calcium levels despite high ROS levels. Ansari et al. [36] found that quercetin and rutin have at least two of these properties: a direct effect on GSH metabolism and ROS scavenger properties [36]. The unsaturated C ring of quercetin confers an additional feature for this flavonoid with regard to its neuroprotective effect [37]. Other authors have demonstrated that quercetin decreases lipid peroxidation, with a reversal in the decrease in GSH and its dependent enzymes and a decrease in catalase and superoxide dismutase (SOD) levels. This antioxidant property was associated with the ability of quercetin to significantly reverse age-related and chronic ethanol-induced retention deficits in cognitive performance in mice [38,39].

In the present study, we verified that oxaliplatin induced a marked lipid peroxidation. Nitrotyrosine immunostaining was also shown to increase in oxaliplatin-induced neuropathy. The effect on NP-SH levels revealed a tendency toward a decrease, but the statistical analyses did not reveal a significant effect. Both rutin and quercetin had antioxidant effects, reflected by inhibition of the lipid peroxidation process and a decrease in nitrotyrosine immunostaining in the dorsal horn of the spinal cord.

Peroxynitrite is a powerful oxidant formed by the reaction between NO and superoxide, and it is more reactive than its precursors. Peroxynitrite reactions lead to the formation of nitrogen dioxide and hydroxyl radicals that directly react with target molecules, mostly tyrosine and lipids. This can be identified in tissue by the formation of nitrotyrosine and lipid peroxidation [40]. In a recent publication, Kumar et al. [41] showed that peroxynitrite and ROS, generated by excessive glutamate, were responsible for neuronal cell death in the rat cerebral cortex [41]. Furthermore, these authors demonstrated that bolus glutamate administration increased iNOS and neuronal nitric oxide synthase (nNOS) mRNA. In the early stage of neurotoxicity, a rapid increase in intracellular Ca^2+^ occurs, with ROS generation and a decrease in GSH. This leads to alterations in nNOS and iNOS mRNA levels with the generation of NO and toxic ONOO- that undergo chemical reactions with various substrates, such as tyrosine nitration and lipid peroxidation [41].

Our data showed that rutin and quercetin inhibited iNOS expression in the dorsal horn region. The protective effect on oxaliplatin neurotoxicity was at least partially attributable to this action. Flavones inhibited the production of NO [42], and quercetin and rutin inhibited LPS-induced iNOS gene expression, reflected by Western blot analysis, in macrophage cultures [43]. Additionally, quercetin, through its potent antioxidant effect, prevented cisplatin-induced cytotoxicity in LLC-PK1 cells in vitro and tubular injury induced by acute renal ischemia in vivo and inhibited LPS-induced NO production [44,45].

In summary, the present study showed that oxaliplatin induced painful peripheral neuropathy in mice, an effect prevented by rutin and quercetin. The oxaliplatin-related neurotoxic effect appears to occur at least partially through oxidative stress-induced damage in dorsal horn neurons, reflected by lipid peroxidation and protein nitrosylation. Lipid peroxidation and tyrosine nitrosylation were prevented by flavonoid treatment. The decreased iNOS expression in the flavonoids-treated groups seems to involve the inhibition of peroxynitrite-associated neuronal damage due to the direct antioxidant effect of these compounds. The present data may lead to a better understanding of the pathogenesis of oxaliplatin-induced neuropathy and the development of new approaches to treat this condition, which includes the prevention of oxidative damage.

## Material and methods

### Animals

The experiments were performed with male Swiss mice (25-35 g) from our own animal facilities in accordance with the local guidelines on the welfare of experimental animals and with the approval of the Ethics Committee in Animal Research of the Federal University of Ceará. The animals were housed in temperature-controlled rooms and received water and food ad libitum. We followed International Association for the Study of Pain Committee for Research and Ethical Issues guidelines for animal research [46]. The researchers who performed the behavioral studies were blinded with regard to treatment.

### Oxaliplatin-induced sensory neuropathy: experimental protocol

Oxaliplatin was diluted in a 5% glucose solution (final concentration: 1 mg/10 mL) and injected intravenously in the lateral vein of the tail at a dose of 1 mg/kg twice a week (on Mondays and Thursdays) with a total of nine injections. The day oxaliplatin was first injected was considered as experimental day 1. The dose used was based on the work of Ling et al. [21] and adapted to our experimental conditions. To evaluate the effect of rutin and quercetin, the mice received an intraperitoneal injection of saline (10 mL/kg), rutin (25, 50, and 100 mg/kg) or quercetin (25, 50, and 100 mg/kg) 30 min before every oxaliplatin injection (1 mg/kg). The oxaliplatin, rutin and quercetin administration stopped after the 9th injection, but the nociceptive tests were conducted once a week up to day 56. Biochemical, histopathology and immunohistochemistry of L4-5 lumbar spinal cord were also performed.

### Behavioral tests

#### Von Frey test

Mechanical nociceptive threshold was assessed by stimulating the hind paws with a pressure meter that consisted of a handheld force transducer fitted with a 0.5 mm^2^ polypropylene tip (electronic von Frey Digital Analgesymeter, Insight Instruments, São Paulo, SP, Brazil). Briefly, in a quiet room, the mice were placed in acrylic cages (12x20x17 cm) with wire grid floors 15–30 min before the start of the test. A tilted mirror placed under the grid provided a clear view of the hind paw. The investigator was trained to apply the tip perpendicularly to the central area of the hindpaw with a gradual increase in pressure. The stimulus was automatically discontinued and its intensity recorded when the paw was withdrawn. The end-point was characterized by the removal of the paw in a clear flinch response after paw withdrawal. The animals were tested before and after treatments. The stimulation of the paw was repeated until the animal presented two similar measurements. If the results were inconsistent (i.e., a marked difference in the baseline response compared with the other animals of the experiment), then the animal was excluded from the study [47].

#### Tail immersion test

Thermal threshold was assessed using the tail immersion test at a non-noxious temperature of 10°C [21,48]. The tail was immersed in the water until the tail was withdrawn. The duration of tail immersion was recorded, with a cut-off time of 15 s. The mice were habituated to the testing procedures and handling by the investigator for 1 week prior to the experiment.

### Histopathological analysis and immunohistochemistry

The animals were deeply anesthetized with ketamine and xylazine (100 mg/kg and 10 mg/kg body weight, respectively, i.p.) and intracardially perfused with freshly prepared 4% paraformaldehyde in 0.1 M phosphate-buffered saline (PBS; pH 7.4). After perfusion, the L4-5 lumbar spinal cord and hind paw skin were removed, post-fixed in the same fixative for 1 h at 4°C, and transferred to 70% ethanol. Transversal sections of the lumbar spinal cord were paraffin-embedded, serially sectioned at a thickness of 4 μm with a microtome, and mounted on slides. For the histopathological analysis, the sections were deparaffinized and hydrated for hematoxylin-eosin staining. The dorsal horn of spinal cord was evaluated in histopathological analysis.

For immunohistochemistry, the sections were deparaffinized and hydrated as similar to above. Hydrated sections were microwaved in 10 mM citrate buffer (pH 6.1) to unmask antigenic epitopes for 15 min at 98°C and allowed to cool for 20 min at room temperature. The sections were washed for 5 min in PBS. Afterward, the sections were hooped with a hydrophobic pen (Dako pen®), incubated for 15 min in peroxidase-blocking solution (3% hydrogen peroxide), and washed for 5 min in PBS. The sections were then incubated with primary polyclonal goat anti-Fos, goat anti-nitrotyrosine (Millipore, USA), or rabbit anti-iNOS antibody (Santa Cruz Biotechnology, Santa Cruz, CA, USA) at 4°C overnight. The sections were then washed in PBS for 5 min and incubated for 30 min at room temperature with biotinylated goat or rabbit secondary antibody (Santa Cruz Biotechnology). After another wash in PBS, the tissue sections were incubated with AB enzyme reagent (streptavidin; reagent A, Avidine; reagent B, biotinylated horseradish peroxidase; Santa Cruz Biotechnology) for 30 min. After a final wash in PBS, the tissue sections were covered with chromogen diaminobenzidine (Liquid DAB + Substrate Chromogen System, Dako North America, Carpinteria, CA, USA) for 2 min and then rinsed twice in distilled water for 5 min. The sections were dehydrated, mounted in a rapid mounting medium (Entellan, Merck, Dannstadt, Germany), and examined with a Leica microscope coupled with DFC232 camera (Wetzlar, Germany). After focusing the microscope, the dorsal horn was photographed at 400× magnification. The quantification of the positive area stained in the pictures was made differentiating the areas (pixels) with higher color saturation associated to immunostaining (brown). For this, we used the program Image J - NIH. The procedure was based on color saturation associated with positive staining for a particular marker. The limits required for defining selected pixels and unselected were previously defined by color threshold. Measuring the total area of photograph and the selected pixels areas it was calculated the percentage of positive stained area in each photograph. It was evaluated, at least 4 slides per group.

### Malondialdehyde assay

The mice subjected to oxaliplatin-induced neuropathy were treated with rutin and quercetin. They were then euthanized by high-dose anesthetics, and the L4-5 lumbar spinal cord was removed. The tissues were immediately frozen in liquid nitrogen and stored at -80°C until use. Lipid peroxidation was determined by measuring MDA production using the Thiobarbituric Acid Reactive Substances (TBARS) test [49]. Briefly, 250 μl of 10% homogenate of tissue sample plus 1.5 ml of 1% H_3_PO_4_ and 0.5 ml of 0.6% thiobarbituric acid aqueous solution were stirred and heated in boiling water for 45 min. After cooling, 2 ml of *n*-butanol was added and stirred, and the butanol layer was collected by centrifugation. The optical density of the *n*-butanol layer was determined at 535 and 520 nm. The difference in optical density between both determinations was calculated and considered the thiobarbituric acid value. Malondialdehyde concentrations are expressed as nanomoles per gram of tissue.

### Statistical analysis

The behavioral data were analyzed using one-way analysis of variance (ANOVA) followed by the Student Newman-Keuls test to detect differences between each treatment and control group at each time point. The data are expressed as mean ± standard error of the mean (SEM). The level of significance was *p* < 0.05.

## Abbreviations

DTNB: 5,5′-dithiobis-(2-nitro-benzoic acid); GSH: Glutathione; i.v.: Intravenous; IB4: Isolectin B4; iNOS: Inducible nitric oxide synthase; LPS: Lipopolysaccharide; MDA: Malondialdehyde; nNOS: Neuronal nitric oxide synthase; NP-SH: Non-protein sulfhydryl group; PBS: Phosphate-buffered saline; ROS: Reactive oxygen species; SOD: Superoxide dismutase; TBARS: Thiobarbituric acid reactive substances; TCA: Trichloroacetic acid.

## Competing interests

The authors declare that they have no competing interests.

## Authors’ contributions

FER performed the behavioral studies. DVW and RPL were responsible for the MDA and NP-SH assays. AFP and RBN per formed the spinal cord dissections and immunohistochemical staining. GAB performed the histopathological analysis and applied the immunohistochemistry scores. MIA performed the behavioral studies, coordinated the experiments, and finished the final draft of the manuscript. MLV and RAR conceived the study, participated in its design and coordination, and helped draft the manuscript. All of the authors read and approved the final manuscript.
